# Bone marrow mesenchymal stem cell‐conditioned medium facilitates fluid resolution via miR‐214‐activating epithelial sodium channels

**DOI:** 10.1002/mco2.40

**Published:** 2020-11-12

**Authors:** Yan Ding, Yong Cui, Yapeng Hou, Hongguang Nie

**Affiliations:** ^1^ Department of Stem Cells and Regenerative Medicine College of Basic Medical Science China Medical University Shenyang China; ^2^ Department of Anesthesiology the First Affiliated Hospital of China Medical University Shenyang China

**Keywords:** acute lung injury, alveolar fluid clearance, epithelial sodium channel, mesenchymal stem cell‐conditioned medium, miR‐214

## Abstract

Acute lung injury (ALI) is featured with severe lung edema at the early exudative phase, resulting from the imbalance of alveolar fluid turnover and clearance. Mesenchymal stem cells (MSCs) belong to multipotent stem cells, which have shown potential therapeutic effects during ALI. Of note, MSC‐conditioned medium (MSC‐CM) improved alveolar fluid clearance (AFC) in vivo, whereas the involvement of miRNAs is seldom known. We thus aim to explore the roles of miR‐214 in facilitating MSC‐CM mediated fluid resolution of impaired AFC. In this study, AFC was increased significantly by intratracheally administrated MSC‐CM in lipopolysaccharide‐treated mice. MSC‐CM augmented amiloride‐sensitive currents in intact H441 monolayers, and increased α‐epithelial sodium channel (α‐ENaC) expression level in H441 and mouse alveolar type 2 epithelial cells. Meanwhile, MSC‐CM increased the expression of miR‐214, which may participate in regulating ENaC expression and function. Our results suggested that MSC‐CM enhanced AFC in ALI mice in vivo through a novel mechanism, involving miR‐214 regulation of ENaC.

## Graphical abstract

Potential mechanisms for MSC‐CM to rescue LPS‐induced lung epithelial injury. MSC‐released paracrine may moderate lung edema caused by LPS via: (1) restoring miR‐214 level; (2) normalizing α‐ENaC expression; (3) recovering ENaC activity; and eventually (4) facilitating edema fluid resolution.

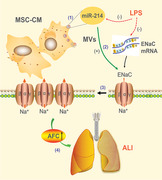

**Abbreviations**
AFC: alveolar fluid clearance
ALI: acute lung injury
ARDS: acute respiratory distress syndrome
AT2: alveolar type 2 epithelial cells
ENaC: epithelial sodium channel
*I*_sc_
: short‐circuit current
LPS: lipopolysaccharide
MSC‐CM: MSC‐conditioned medium
MSCs: mesenchymal stem cells

## 1 INTRODUCTION

Acute lung injury (ALI) and acute respiratory distress syndrome (ARDS) are both common and multifactorial clinical syndromes, which are characterized by acute onset, increased pulmonary permeability, impaired alveolar microvascular endothelium and alveolar epithelium, as well as severe lung edema.^1^, ^2^ Most current therapies of ALI are still supportive through decades of intensive research exploration, thus there is an urgent need to explore novel and more effective interventions.^3^, ^4^ The process of alveolar fluid clearance (AFC) performed in alveolar epithelial cells involves epithelial sodium channel (ENaC), aquaporin and Na^+^/K^+^‐ATPase under physiological conditions and lung edema, among which ENaC is major determinant pathway in forming the osmotic sodium gradient over the pulmonary epithelium.^5^, ^6^, ^7^, ^8^, ^9^, ^10^, ^11^

Mesenchymal stem cells (MSCs) are nonhematopoietic stem‐like cells first discovered from the niche of bone marrow, capable of multilineage differentiation, immune regulation, and tissues repair.^12^, ^13^, ^14^ Recent studies showed that MSCs have the potential to alleviate the degree of lung injury in diverse disease models through paracrine effects, especially via the carrier of MSC secretome, MSC‐conditioned medium (MSC‐CM).^15^, ^16^, ^17^ Previous studies often focused on cell‐based therapy. However, we have to overcome some obvious hurdles if MSCs are administrated directly in clinical tests, including the provision for numerous MSCs, the deficiency of MSCs quality, and the continuous differentiation of MSCs after in vitro passaging.^18^

Most precious studies focused on the inflammation moderation, tracking to impaired tissues, and the attenuation of lung permeability by MSCs in ALI/ARDS therapy, whereas the mechanisms of MSC‐CM in restoring lung edema, especially how MSC‐CM modulated the ENaC‐related fluid transport in alveolar epithelium was rarely reported.^19^, ^20^, ^21^ MSCs have the ability to regulate the immune response to tissue damage and promote repair in vivo, and have been suggested to act on a variety of lung diseases (including ALI). Of note, MSCs can release lots of cytokines/growth factors, signal lipids, exosomes, and miRNAs. By performing microarray hybridization, Chen et al found at least 151 miRNAs in MSC through testing the RNAs.^22^ We have previously tested several miRNAs, including miR‐124‐5p, miR‐130b, and so on. In our recently published paper, we investigated miR‐124‐5p, which existed in MSC‐CM, and found that the miR‐124‐5p participated in the regulation of MSC‐CM in lipopolysaccharide (LPS)‐induced ALI by targeting α‐ENaC.^23^ Similarly, miR‐214 was found in MSCs and has been reported to exert key roles in various inflammatory diseases, whereas its role in ALI and the relative mechanisms are seldom studied.^24^, ^25^ Therefore, we chose miR‐214 as our main research object, and cultured it with alveolar type 2 epithelial (AT2) cells to explore its role in the regulation of MSC‐CM in LPS‐induced ALI, aiming to provide a novel direction for therapeutic strategy in ALI. MiR‐214 derived from MSC‐CM is a potential novel regulator of inflammation, while its deeper mechanisms in ALI are not clear.^26^ In our study, we attempted to mimic a “cell‐free therapy” method for alleviating the severity of lung edema by administrating MSC‐CM in ALI injured lungs. This potent intervention will avoid the limitations reported in previous studies by utilizing the protective immunomodulatory paracrine factors of MSCs, which may provide a promising therapeutic strategy in ALI.

## 2 MATERIALS AND METHODS

### 2.1 Animals preparation

Four‐week old, 10‐15 g weight male C57 mice (n = 5) were used for isolation of MSC‐CM; 8‐10 weeks, 25‐30 g weight male C57 mice (n = 45) were studied for AFC test. All mice were given a 12 hour light/dark cycle in an air‐conditioned room. AT2 cells were obtained from newborn mice (within 24 hours, n = 20). All specific pathogen‐free mice were purchased from the Laboratory Animal Center in China Medical University. The study was performed according to the regulation of Animal Care and Use Ethics Committee of China Medical University, and the certificate No. is SYXK (Liao) 2018‐0008. Mice were kept without pathogens and with free water/chow access.

### 2.2 Isolation of MSC‐CM and AT2 cells

The male C57 mice were anesthetized by diazepam (17.5 mg/kg) and 6 minutes later by ketamine (450 mg/kg), intraperitoneally. We isolated femora and collected bone marrow by gently washing medullary cavity with DMEM (Dulbecco’s Modified Eagle Medium)/F12 medium (HyClone) containing FBS (10% Gibco), penicillin (100 IU), and streptomycin (100 μg/mL), and recombinant mouse basic fibroblast growth factor (10 ng/mL, PeproTech). The medium was changed after 24 hours to remove nonadherent cells and tissues, and replaced every other day. At 80% confluence, the medium was changed to FBS‐deprived medium, and MSCs‐CM was obtained in 24 hours and stored at −80°C after being filtered with 0.22 μm ultrafiltration membranes.

We separated newborn mouse lung lobes and isolated AT2 cells in cold PBS. Lung tissue was teased and digested with trypsin/collagenase (Sigma) for 0.5 hour, respectively. Cells were cultured in 5% CO_2_, 37°C in DMEM/F12 supplemented with 10% FBS (Fetal Bovine Serum) for 45 minutes. We collected unattached cells and repeated the process four times, removing lung fibroblast cells. The cell suspension was transferred on culture dish coated by IgG for 1 hour to remove the other unnecessary cells. We adjusted unattached cells to 3 × 10^6^/mL, and changed the medium after 72 hours, then replaced every 48 hours.

### 2.3 Drug administration

For in vivo ALI model, the method was modified from previous studies.^27^, ^28^ Eight to ten‐week, 25‐30 g weight male C57 mice were administered LPS (5 mg/kg) or PBS (control group) intraperitoneally. The mice were treated with FBS‐deprived DMEM/F12 medium or MSC‐CM intratracheally after 4 hours, and used in AFC assay 20 hours later.^29^ For in vitro cell models, H441 and AT2 cells were administrated with LPS (10 μg/mL, 12 hours) and/or MSC‐CM (24 hours), respectively.^30^ The control groups were given equivolume PBS (Phosphate Buffer Saline).

### 2.4 Mouse AFC

AFC was performed in vivo as we mentioned earlier.^31^ Briefly, C57 mice were supplied 100% O_2_ via a ventilator (Chengdu Taimeng Co. LTD) after being anesthetized by 17.5 mg/kg diazepam and 450 mg/kg ketamine intraperitoneally. Five percent bovine serum albumin (BSA) (200 μL) solution with/without amiloride (1 mM, Sigma) was instilled intratracheally, and 30 minutes later the alveolar fluid was collected. AFC was calculated as the following formula: AFC = (Vi − Vf)/Vi × 100, in which Vi and Vf indicate the volume of the alveolar fluid instilled and collected, respectively. Vf = (Vi × Pi)/Pf, in which Pi and Pf are concentrations of BSA in the instilled and collected fluid, respectively. The mean and SE values of amiloride‐sensitive AFC (AS AFC) fraction were calculated^32^:

$$\mathrm{Mean}=\mathrm{Mt}-\mathrm{Ma}\pm \mathrm{tc}\times \mathrm{SE}\times \sqrt{\frac{1}{n_{t}}+\frac{1}{n_{a}}}$$

In the above equation, Mt and Ma represent the mean AFC value of total and amiloride‐resistant fraction, respectively; and tc signifies the *t*_95_ value for the free‐dome of (*n*_t_ + *n*_a_ − 2). SE and *n* indicate the standard error and mouse number, respectively.

### 2.5 H441 monolayers

H441 cells, an epithelial cell line from human distal lung, were acquired from the ATCC, and cultured as described previously.^23^, ^33^ For Ussing chamber assay, cells (∼5 × 10^6^ cells/cm^2^) were grown on Costar Transwells (Corning‐Costar, Lowell, MA, USA) for 24 hours until reaching confluence. Then, cells were cultured in air‐liquid interface model, and the basolateral compartment medium was replaced every 48 hours. The short‐circuit current (*I*_sc_) was measured in the confluent monolayers with resistance >500 Ω cm^2^, which was checked by an epithelial tissue volt‐ohm meter (WPI, Sarasota, FL, USA).

### 2.6 Ussing chamber measured

Transepithelial *I*_sc_ and resistance were carried out in H441 monolayers as described previously.^34^ Briefly, cell monolayers were installed in Ussing chamber setup (Physiologic Instruments, San Diego, CA, USA) and immersed into a 37°C conductive solution supplied with 95% O_2_ and 5% CO_2_. The pH and osmolality were controlled to 7.4 and 290‐300 mOsm/kg, respectively. We clamped the monolayers to 0 mV, and applied a 10 mV pulse with 1 second duration to monitor the short‐circuited resistance of transepithelium. The data of *I*_sc_ were acquired from Acquire and Analyze 2.3 program, and the activity of ENaC was the reduction of *I*_sc_ induced by amiloride (100 μM).

### 2.7 Western blot assay

The protein lysates were separated in 10% SDS‐PAGE gels and then transferred to PVDF membranes (Invitrogen). Western blot assay was performed with first antibodies to α‐ENaC (PA1‐920A, Thermo Fisher), β‐actin (sc‐47778, Santa Cruz Biotechnology) at 1:2000, and goat‐anti‐rabbit/mouse second antibody at 1:2000. Blots were probed with the ECL kit and obtained by Image J program. The specific α‐ENaC protein band was seen between 70 and 100 kDa, in accordance with our previous studies.^35^

### 2.8 Real‐time polymerase chain reaction

We isolated total RNA using TRIzol (Invitrogen), and the concentration was measured by NanoDrop 2000c spectrophotometry (Thermo). Total RNA/miRNA reverse transcription was performed using PrimeScript RT reagent Kit and Mir‐X miRNA First‐Strand Synthesis Kit (TaKaRa). Real‐time polymerase chain reaction (RT‐PCR) was performed with SYBR Premix Ex Taq II (TaKaRa) using ABI 7500 qRT‐PCR System with the primers against α‐ENaC forward, 5′‐AAC AAA TCG GAC TGC TTC TAC‐3′; reverse, 5′‐AGC CAC CAT CAT CCA TAA A‐3′; and GAPDH forward, 5′‐AGA AGG CTG GGG CTC ATT TG‐3′; reverse, 5′‐AGG GGC CAT CCA CAG TCT TC‐3′. After normalizing data to GAPDH/U6, the relative expressions to control groups were analyzed by the 2^−△△CT^ method.

### 2.9 Transfections

Cells were transfected with negative control and miR‐214 mimic (30 nM, Genepharma) with siRNA‐mate (Genepharma, Suzhou, China) for 6 hours, and used 48 hours posttransfection.

### 2.10 Statistical analysis

Data were presented as the mean ± SE. Firstly, the power of sample size were evaluated to meet *P* < .05. Normality and homoscedasticity analyses were applied next by Levene and Shapiro‐Wilk before conducting parametric tests. We used Student's two‐tailed *t*‐test for comparing two groups; as for the comparison among multiple groups, variance (ANOVA) was performed, and Bonferroni's test was followed. When the data did not satisfy the applied condition of the normality/homoscedasticity test, we tried nonparametric *t*‐test (Mann‐Whitney U test) instead. *P* < .05 was considered significant variation, and the statistical analysis was performed by Origin 8.0.

## 3 RESULTS

### 3.1 MSC‐CM enhances mouse AFC in vivo

**FIGURE 1:**
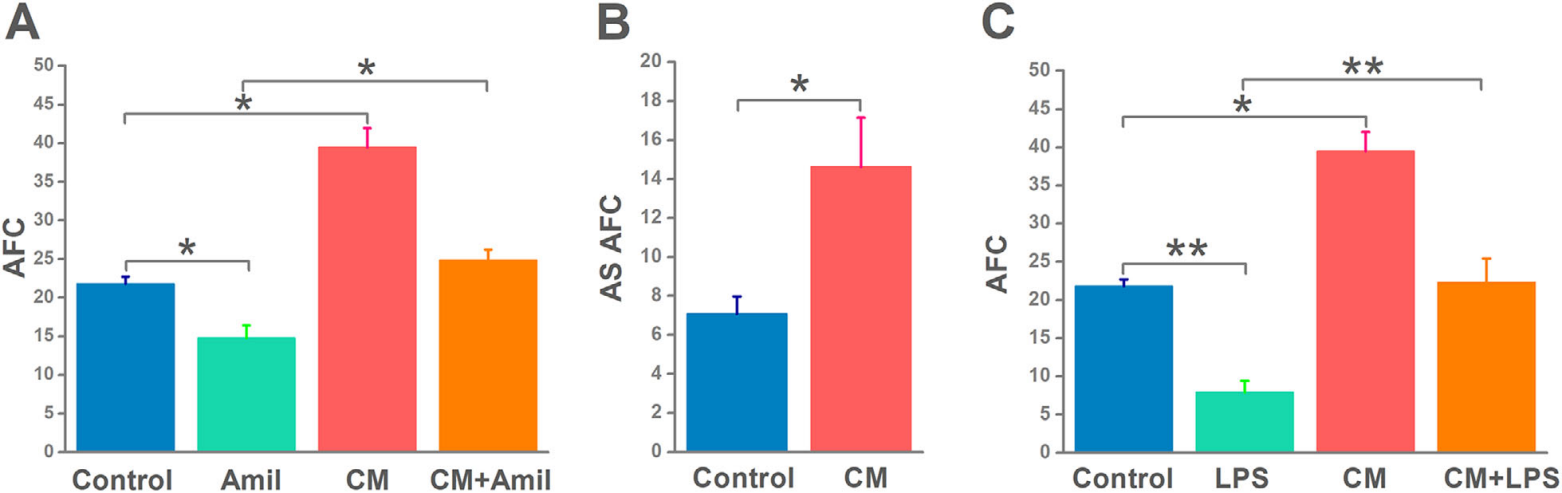
Upregulation of mouse alveolar fluid clearance (AFC) in vivo by mesenchymal stem cell‐conditioned medium (MSC‐CM). A, Intratracheal application of MSC‐CM increases AFC. Mice were pretreated with FBS‐deprived DMEM/F12 medium or MSC‐CM intratracheally for 20 hours. In AFC assay, mice were intratracheally instilled with 5% BSA dissolved in FBS‐deprived DMEM/F12 medium alone for the control/CM group or containing amiloride (Amil/CM + Amil group). B, Computed amiloride‐sensitive AFC (AS AFC) associated with ENaC. C, MSC‐CM restores LPS‐reduced depressed AFC in mice treated with LPS (5 mg/kg, 24 hours, intraperitoneally). n = 5‐6. *P < .05; **P < .01

To investigate the influence of MSC‐CM on the reabsorption of fluid in mouse distal lung, we proposed AFC in vivo in C57 mice by measuring the instillate of 5% BSA. As shown in Figure 1A, mice pretreated with MSC‐CM significantly increased the reabsorption of 5% BSA instillate. In order to clarify the augment of AFC by MSC‐CM is correlated with the activation of ENaC, we then applied a specific inhibitor of ENaC, amiloride, which downregulated AFC significantly. The difference between total and amiloride‐resistant AFC was considered as AS AFC, and MSC‐CM administration markedly increased AS AFC, which reflected ENaC‐mediated fluid transport (Figure 1B). To explore the effects of MSC‐CM on AFC during ALI, we treated mice with LPS for 24 hours. As shown in Figure 1C, MSC‐CM upregulated the LPS suppressed AFC, indicating MSC‐CM may benefit edematous ALI by enhancing ENaC activity.

### 3.2 MSC‐CM increases transepithelial *I*_sc_ in confluent H441 monolayers

**FIGURE 2:**
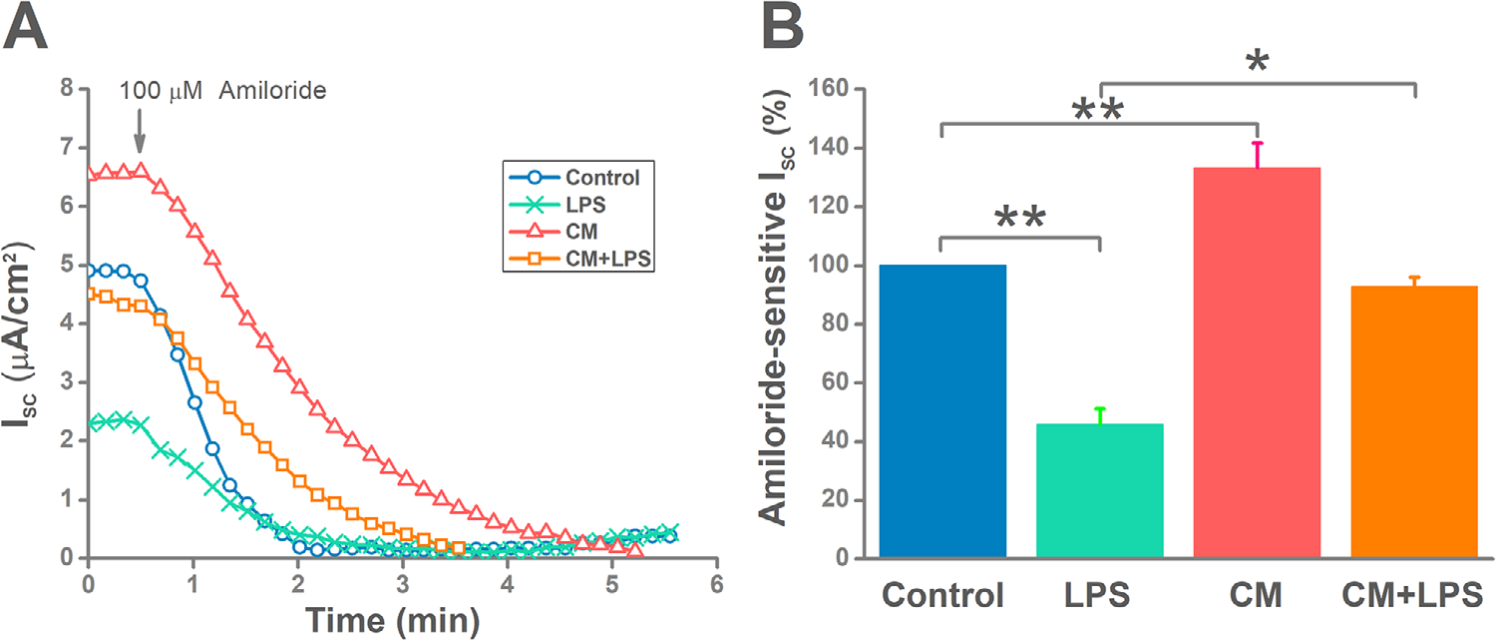
Activation of epithelial sodium channel (ENaC) by mesenchymal stem cell‐conditioned medium (MSC‐CM) in polarized H441 monolayers. A, Representative *I*_sc_ traces measured after H441 monolayers were treated with LPS (15 μg/mL) for 12 hours and/or MSC‐CM for 24 hours. B, Average amiloride‐sensitive *I*_sc_ in control group, LPS group, CM group, and (CM + LPS) group. n = 5. *P < .05; **P < .01

We next investigated the ENaC function in the lung using H441 monolayers, as the ENaC properties of Clara cells (H441) from human bronchoalveolar epithelia are alike with those of primary AT2 cells.^32^, ^36^, ^37^ To further confirm ENaC activity was regulated by MSC‐CM, we recorded *I*_sc_ in H441 monolayers (Figure 2A). The *I*_sc_ at time 0 was the total current measured in H441 monolayers, which reflected the activities of both apical and basolateral channels/transporters. The remaining *I*_sc_ after adding amiloride was the fraction that is amiloride‐resistant, while the amiloride‐sensitive *I*_sc_ (ASI, %) was calculated as the total *I*_sc_ minus amiloride‐resistant *I*_sc_, then divided by the initial ASI (×100), reflecting the ENaC activity. As shown in Figure 2B, MSC‐CM abrogated the inhibition effect of LPS on ASI. Our results illustrated that MSC‐CM could not only increase the total current, but also ASI, which is mainly mediated by ENaC.

### 3.3 MSC‐CM increases α‐ENaC protein expression in H441 and AT2 cells

**FIGURE 3:**
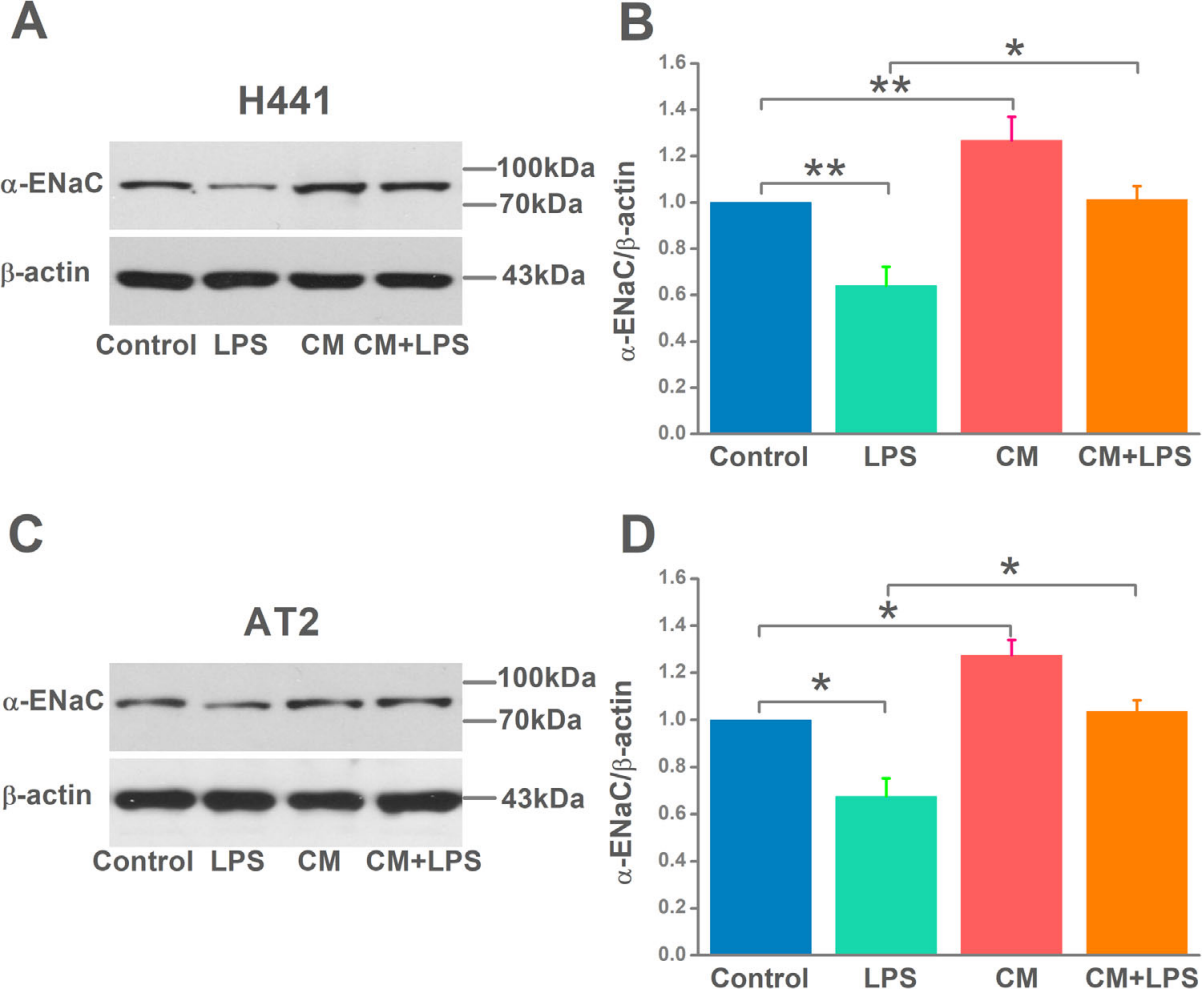
Mesenchymal stem cell‐conditioned medium (MSC‐CM) increases α‐epithelial sodium channel (α‐ENaC) protein level in H441 and alveolar type 2 epithelial (AT2) cells. A and C, Representative western blot measurement of α‐ENaC from H441 and AT2 cells treated with LPS (15 μg/mL) for 12 hours and/or MSC‐CM for 24 hours. B and D, Semi‐quantitation of western blots (α‐ENaC/β‐actin). n = 4‐5. *P < .05; **P < .01

In view of the evidence of activated ENaC function shown in AFC and *I*_sc_, we future examined the effects of MSC‐CM on ENaC expression at protein levels in H441 and AT2 cells. The α‐ENaC, one of ENaC subunits, presents a critical role required for Na^+^ transport. As shown in Figure 3, we observed that LPS stimulated a significant downregulation in α‐ENaC protein expression, whereas administration with MSC‐CM enhanced α‐ENaC expression in H441 cells. Similar results were also observed in AT2 cells. We therefore proposed that MSC‐CM restored AFC and *I*_sc_ reduced by LPS may be associated with the enhanced expression level of α‐ENaC protein.

### 3.4 MSC‐CM augments transcription levels of α‐ENaC and miR‐214 in AT2 cells

**FIGURE 4:**
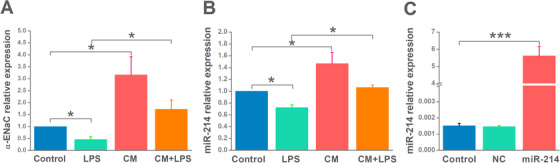
Upregulation of miRNA‐214 and α‐epithelial sodium channel (α‐ENaC) transcripts by mesenchymal stem cell‐conditioned medium (MSC‐CM) in alveolar type 2 epithelial (AT2) cells. The mRNA samples for RT‐PCR assays were isolated from AT2 cells treated with LPS (15 μg/mL) for 12 hours and/or MSC‐CM for 24 hours. A, The expression level of α‐ENaC mRNA was examined with GAPDH set as an internal standard. B‐C, The relative level of miR‐214 was calculated with U6 set as an internal standard. n = 4. *P < .05; ***P < .001

In the view of MSC‐CM increasing the protein expression of ENaC, we speculated that MSC‐CM might increase mRNA expression of ENaC, which provide a template for protein synthesis. The results showed that α‐ENaC transcription level in LPS‐treated AT2 cells was markedly increased after MSC‐CM administration (Figure 4A).

Previous studies have suggested that miR‐214 derived from MSCs and MSC‐CM is the regulator of ERK1/2, which is related with ENaC.^22^, ^38^, ^39^, ^40^ We speculate that MSC‐CM can upregulate miR‐214 level, and then activate α‐ENaC expression in AT2 cells. Consistent with this hypothesis, we found a lower miR‐214 level in ALI in vitro model compared with control group, whereas a higher level of miR‐214 was observed after administration of MSC‐CM (Figure 4B), showing that MSC‐CM may moderate LPS‐induced ALI through increasing the miR‐214 expression level.

### 3.5 MiR‐214 increases α‐ENaC protein expression and ASI

**FIGURE 5:**
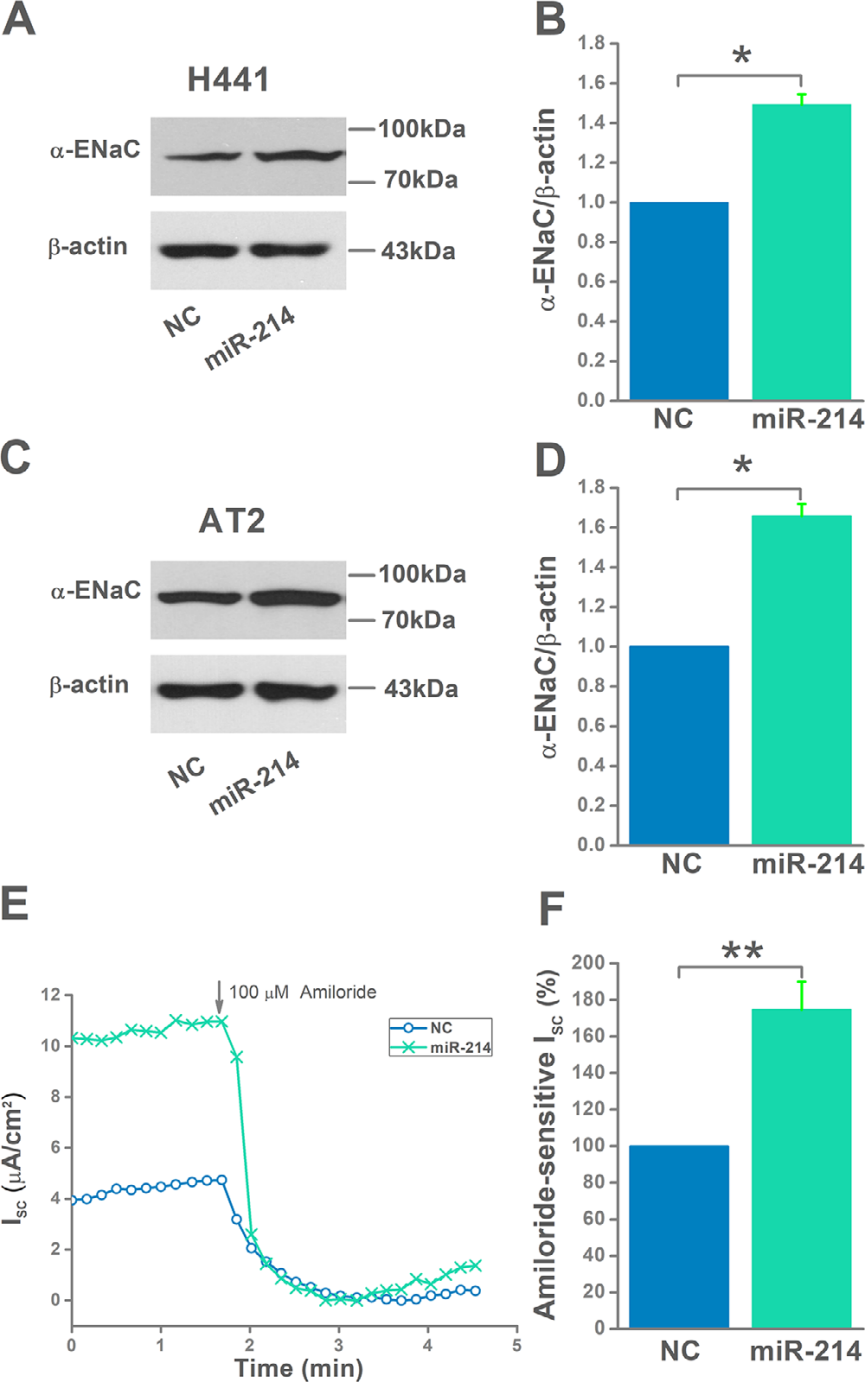
Activation of epithelial sodium channel (ENaC) by mesenchymal stem cell‐conditioned medium (MSC‐CM) through regulating miR‐214 levels. A and C, Representative western blot measurement of α‐ENaC protein in H441 and AT2 cells transfected with miR‐214 negative control (NC) or miR‐214 mimic. B and D, Representative graphical representation of α‐ENaC expression in H441 and AT2 cells, n = 4. E, Representative *I*_sc_ traces recorded in H441 monolayers transfected with NC or miR‐124 mimic. F, Average amiloride‐sensitive *I*_sc_. n = 6. *P < .05; **P < .01

Based on the higher miR‐214 expression in AT2 cells after MSC‐CM administration, we postulate that miR‐214 has a positive effect on α‐ENaC expression and activity, which are key mediators of MSC‐CM in ENaC‐associated lung edema. We first transfected H441 and AT2 cells with miR‐214 mimic/negative control, which were next used in western blot and Ussing chamber assay to measure α‐ENaC expression and activity, respectively. The result showed that miR‐214 expression was markedly enhanced after transfection with the miR‐214 mimic (Figure 4C). Transfection of miR‐214 mimic not only led to a significant upregulation of α‐ENaC expression in H441 and AT2 cells, but also ASI in confluent H441 monolayers (Figure 5).

## 4 DISCUSSION

Despite the gradually extensive application of MSC‐based therapy in clinic at present, there are still challenges that need to be valued, such as the clarification of MSC phenotypes, quantifying the effects of MSCs, and concerns about the safety and efficacy.^18^ The beneficial effects of MSCs are owing to the transient secretion of biologically active regenerative and immunomodulatory factors.^41^ With that knowledge, an effort has been made to analyze beneficial effects of cell‐free secreted factors in different diseases. MSC‐CM as a cell‐free preparation has several apparent advantages over cell therapy, which has thrombogenic potential as well as other less thought of risks such as arrhythmia, ossification, and calcification.^42^, ^43^ In addition, cryopreservation may yield MSCs with reduced viability and function in vivo.^44^ All these effects and concerns can be avoided with MSC‐CM, which rarely requires expensive infrastructure for the extraction of autologous cells and/or ex vivo expansion of both autologous and allogeneic cells. Therefore, a cell‐free treatment strategy based on the paracrine effects will avoid the indeterminacy of MSC‐based therapies, and offer a more attractive platform to overcome above challenges. Of note, microvesicles (MVs) derived from MSCs have been described as important mediators of intercellular communication between stem cells and other cells, which contain numerous bioactive substances including the miRNAs. MiR‐214‐containing MVs from MSCs and MSC‐CM can be delivered into injured cells via paracrine effects and regulate recipient cells accordingly.^22^

ALI/ARDS is featured by impaired alveolar epithelium and lung endothelium resulting in increased lung permeability, lung edema, alveolar space filling, and respiratory insufficiency at the early phase. Substantial early clinical studies have confirmed that the increased function of AFC process is essential for the resolution of lung edema in ALI,^7^, ^8^, ^45^, ^46^ which is mediated by ion channels, containing the ENaC.^47^ Among the three main subunits, α‐ENaC is critical for channel functionality and the mice knocked out α‐ENaC gene died for the insufficient AFC during the perinatal period, while β and γ ENaC are regulatory subunits and can maximize the channel function.^48^, ^49^

Many studies reported the therapeutic effects of MSCs on ALI through cytokines; however, information on the exact roles of MSC‐CM‐derived miRNAs in lung edema during ALI and the relative mechanisms of altered ion transport are still scarce.^50^, ^51^, ^52^, ^53^ In our study, we explored a cell‐free therapy method and investigated the impacts of MSC‐CM on edematous lung injury. In the lung epithelial cells comprising alveolar epithelium, AT2 cells can produce surfactant, and exert a role in body defense and transepithelial transport.^23^, ^54^, ^55^ The AT2 cell model in ALI was commonly constructed in previous studies, and we explored the mechanism of MSC‐CM facilitating fluid resolution via miR‐214 activating ENaC in primary AT2 cells, which mainly express ENaC and work in fluid and ion transport in ALI. Therefore, we used primary AT2 cells in our in vitro experiments.

The amiloride‐sensitive AFC and *I*_sc_, which reflected ENaC activity, were both increased by MSC‐CM, suggesting the potential effects of MSC‐CM in lung edema may be relative to the activation of ENaC. MSC‐CM has been reported to be administrated by the route of intraperitoneal, intratracheal, and intravenous injection. In our experiment, we chose intratracheal administration route of MSC‐CM and studied its effect on the lungs based on the following reasons. First, intratracheal administration enables that medication targets effectively to the injured lung, resulting in better treatment outcomes and requiring less dosage than other administration routes. Second, intratracheal administration exerts effect as quickly as intravenous injection, which is faster than other administration routes. Finally, intratracheal administration can avoid low bioavailability, unnecessary metabolism, and so on. Furthermore, the evidence was also supported by our molecular level observation that the protein and mRNA expression of ENaC were subsequently upregulated after administration of MSC‐CM in H441 and AT2 cells, indicating that MSC‐CM increased the ENaC‐mediated fluid transport in LPS‐treated alveolar epithelial cells via enhancing α‐ENaC expression.

**FIGURE 6:**
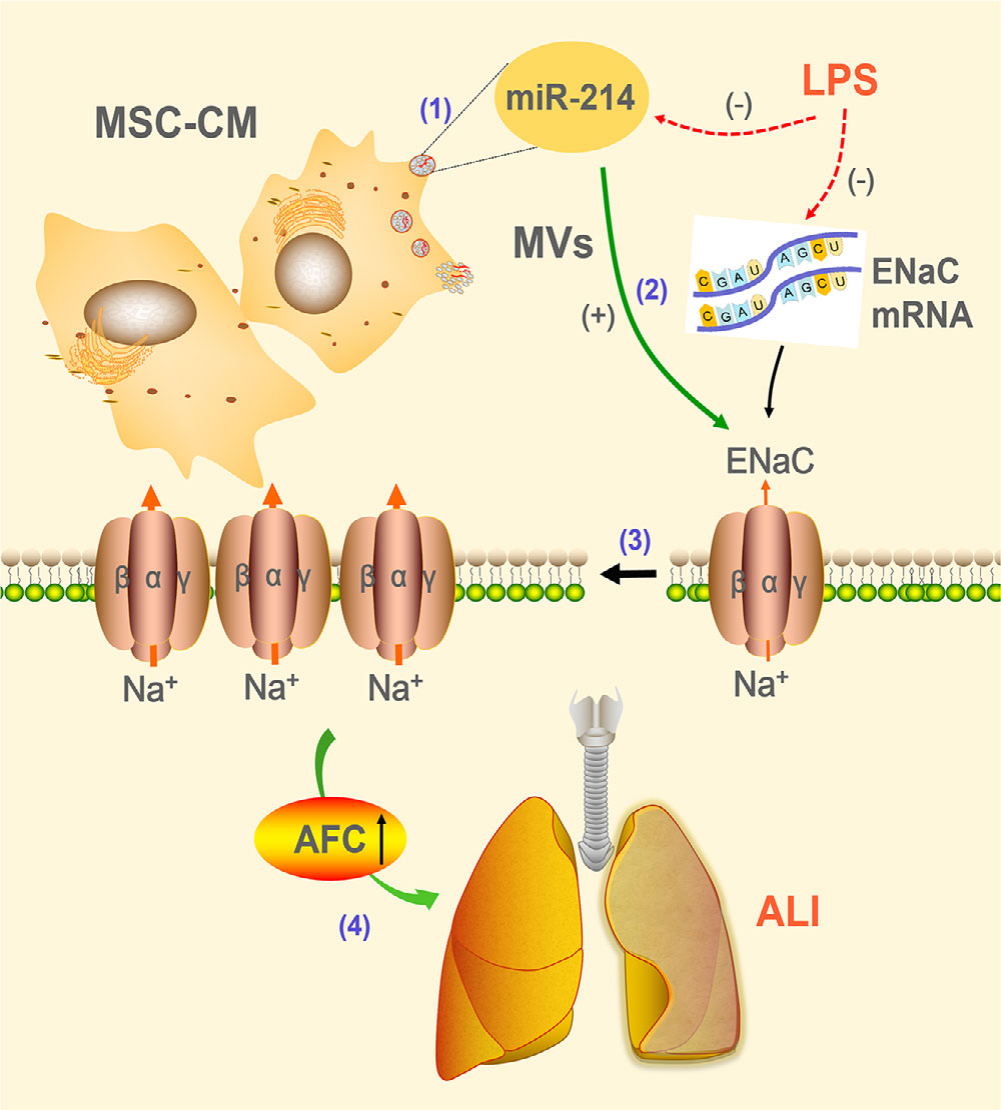
Potential mechanisms for mesenchymal stem cell‐conditioned medium (MSC‐CM) to rescue lipopolysaccharide (LPS)‐induced lung epithelial injury. MSC‐released paracrine may moderate lung edema caused by LPS via: (1) restoring miR‐214 level; (2) normalizing α‐ENaC expression; (3) recovering ENaC activity; and eventually (4) facilitating edema fluid resolution

To identify the effects of miR‐214 in the MSC‐CM‐regulated α‐ENaC activity, we ascertained the miR‐214 expression in AT2 cells; higher miR‐214 levels found in LPS‐treated cells after administration of MSC‐CM. As expected, we found that miR‐214 significantly increased both α‐ENaC protein expression and ASI of alveolar epithelial cells, identifying the activation of miR‐214 on MSC‐CM‐regulated ENaC. Previous studies have shown that miR‐214 can potentially target phosphatase and tensin homolog (PTEN), which negatively regulates PI3K.^56^, ^57^, ^58^, ^59^ The activating PI3K/Akt pathway may inhibit ENaC degradation from plasma membrane through Nedd4‐2 or SGK1 pathway, contributing a better understanding of miR‐214 upregulating ENaC.^60^, ^61^, ^62^ These data suggested that MSC‐CM may transfer MVs containing miR‐214 to recipient cells, which enhances ENaC function by promoting the protein expression level of α‐ENaC, so as to enhance AFC and exert a beneficial effect in LPS‐induced ALI (Figure 6). However, the reason why MSC‐CM upregulated the expression level of miR‐214 is still a problem that awaits our future investigation. We will next focus on the regulation of miR‐214 on ENaC in ALI in the in vivo and in vitro models and the underlying mechanisms of MSC‐CM on the miR‐214‐related beneficial effects in LPS‐induced ALI.

## 5 CONCLUSION

Taken together, our findings illustrated that MSC‐CM restored the depressed α‐ENaC expression and activity during LPS‐induced ALI, at least partly via enhancing miR‐214.

## AUTHOR CONTRIBUTIONS

Hongguang Nie conceived and designed the study. Yong Cui and Yan Ding performed the study and analyzed the data. Yapeng Hou and Hongguang Nie drafted the manuscript. Yong Cui revised the draft of manuscript. All the authors corrected and approved the final version of the manuscript.

## CONFLICT OF INTEREST

The authors declare that there is no conflict of interest.

## FUNDING INFORMATION

National Natural Science Foundation of China, Grant Number: NSFC 81670010; Provincial Key Research and Development Program Guidance Project, Grant Number: 2018225077; Basic Research Project of Liaoning Higher School, Grant Number: LQNK201745

## Data Availability

Research data are available from the corresponding author upon reasonable request.
